# Generalized quasi-linear approximation and non-normality in Taylor–Couette spiral turbulence

**DOI:** 10.1098/rsta.2022.0122

**Published:** 2023-03-20

**Authors:** Jeffrey S. Oishi, Morgan Baxter

**Affiliations:** Department of Physics and Astronomy, Bates College, Lewiston, ME, USA

**Keywords:** generalized quasi-linear approximation, Taylor–Couette flow

## Abstract

Taylor–Couette flow is well known to admit a spiral turbulence state in which laminar and turbulent patches coexist around the cylinder. This flow state is quite complex, with delicate internal structure, and it can be traced into certain regimes of linear stability. This behaviour is believed to be connected to the non-normality of the linear operator, which is itself a function of the control parameters. Using spiral turbulence in both linearly stable and unstable regimes, we investigate the effectiveness of the generalized quasi-linear approximation (GQL), an extension of quasi-linear theory designed to capture the essential aspects of turbulent flows. We find that GQL performs much better in the supercritical regime than the subcritical. By including only a small number of modes in the nonlinear interactions, GQL simulations maintain a turbulent-like state when in the supercritical regime. However, a much larger number is required to avoid returning to the laminar state when in the subcritical regime.

This article is part of the theme issue ‘Taylor–Couette and related flows on the centennial of Taylor’s seminal *Philosophical Transactions* paper (part 1)’.

## 1. Introduction

Since Taylor’s classic 1923 paper [1], fluid between two independently rotating cylinders has become a rich laboratory for the understanding of instability, transition to turbulence and turbulence itself. Indeed, the interplay between experiment, computation and theoretical understanding evinced in Taylor’s paper remains a model for the study of fluiddynamics.

Some of the most common forms of turbulence in nature are anisotropic, with a symmetry broken by rotation, magnetic fields or boundary effects. In these situations, significant theoretical progress can be made by considering *quasi-linear* (QL) models, in which one retains only those nonlinearities that interact with the mean flow. Recently, the generalized quasi-linear approximation (GQL) has been developed to systematically expand the QL idea to include sets of nonlinear interactions between two *sets* of modes, low and high, respectively (see [2] for a recent review). GQL has been shown to perform significantly better in reproducing direct numerical simulations (DNS) than QL models across a wide range of paradigm flows including forced zonal jet formation [3], rotating plane Couette flow [4], rapidly rotating convection [5] and channel flow [6–8].

Here, we consider Taylor–Couette (TC) flows at moderate inner and outer Reynolds numbers, $|{Re}_{i,o}|<3500$. This is an interesting test bed for GQL because it features numerous intricate patterns that are maintained by nonlinear interactions beyond the cascade, local in coefficient space, found at high Re. One of the key features of TC flow is that it has nonlinear and linear instabilities in different regions of parameter space. In the linearly stable regions, transient growth has been observed in TC flow [9,10]. We can write the Navier–Stokes equations in schematic form as

1.1 $$\frac{\partial \text{q}}{\partial t}+L\text{q}=N(\text{q}),$$

where L is a linear operator and N is a nonlinear function of the state vector $q={\left[u,p\right]}^{T}$, where u is the velocity field and p the pressure. As noted by Hristova *et al*. [9], the non-normality of the linear operator L for TC flow is a function of its control parameters. That is, its commutator with its adjoint

1.2 $$\left[L^{†},L]=f(\mu ,\eta ,{Re}_{i},\Gamma \right)$$

can be zero or non-zero, and f is a function of the four parameters of TC flow: the inner cylinder Reynolds number ${Re}_{i}$, the ratio of inner to outer radii $\eta \equiv R_{i}/R_{o}$, the ratio of outer to inner rotation rates $\mu \equiv \Omega_{o}/\Omega_{i}$ and the aspect ratio of the cylinder $\Gamma \equiv L_{z}/d$. This provides a very useful arena to test the validity of GQL in a system that admits a varying degree of non-normality.

As a first experiment to this end, we consider the *spiral turbulence* flow regime. The classic paper by Coles [11] was the first to note a hysteretic transition to spiral turbulence when the cylinders were counter-rotating: an experiment at fixed ${Re}_{o}$ will have spiral turbulence at a certain ${Re}_{i}$ if ${Re}_{i}$ is reached by *decreasing* from a higher value, but will not have spiral turbulence at the same ${Re}_{i}$ if it is reached by *increasing* from a lower value. More recent work has identified both supercritical and subcritical paths to spiral turbulence [12]. In that work, the authors follow spiral turbulence along a decreasing ${Re}_{i}$ path and show that the state is maintained even below the linear stability boundary when ${Re}_{o}=-3000$ but it relaminarizes below the stability boundary when ${Re}_{o}=-1200$. Using [12] as a guide, we use DNS at ${Re}_{o}=-3398$ and decrease ${Re}_{i}$ into the subcritical region. We then run a suite of GQL simulations in both the super- and subcritical regions.

We begin in §2 by detailing our equations, non-dimensionalization and numerical methods. Section 3 reviews both modal and non-modal linear analyses of spiral turbulence. In §4, we describe the main results of the paper, the comparison of DNS, QL and GQL for supercritical and subcritical spiral turbulence. Finally, we offer discussion and concluding remarksin §5.

## 2 Methods

We solve the incompressible Navier–Stokes equations with unit density for $u^{\prime }=u-U$, where $U=Ar+B/r{\hat{e}}_{\phi }$ is circular Couette flow (CCF) and A and B are the standard constants (see [13], ch. 7),

2.1 $$\frac{\partial \text{u'}}{\partial t}+\text{U}\cdot \nabla \text{u'}+\text{u'}\cdot \nabla \text{U}+\text{u'}\cdot \nabla \text{u'}=-\nabla p+\nu \nabla^{2}\text{u'}$$

and

2.2 ∇⋅u'=0,

where p is the pressure and ν is the kinematic viscosity. We solve equations (2.1) without modification for the DNS runs. To briefly review, GQL extends the quasi-linear approximation by dividing $u^{\prime }$ into two subsets,

2.3 $${\text{u'}}_{l}={\left\langle \text{u'}\right\rangle }_{l}=\sum_{m=-\Lambda_{\theta }}^{\Lambda_{\theta }}\sum_{k=-\Lambda_{z}}^{\Lambda_{z}}{\hat{u}}_{mk}^{\prime }(r)\;e^{i(m\theta +2\pi kz/L_{z})}$$

and

2.4 $${\text{u'}}_{h}=\text{u'}-{\text{u'}}_{l},$$

which we refer to as the ‘low’ and ‘high’ modes, respectively. $\Lambda_{z}$ and $\Lambda_{\theta }$ are adjustable parameters that represent the *cut-off* in wavenumber space delineating low from high. The nonlinearity, when expressed in spectral space, is a triadic interaction between wavenumbers. GQL includes all such interactions that fall into three classes: low–low → low, high–high → low and low–high → high, and it discards all others. This is a closed system that respects all conservation laws of the original equations [3]. Most importantly, it represents a systematic closure of increasing fidelity independent of a particular theoretical model for the underlyingturbulence.

We introduce a projection operator to perform this division, in which case GQL can be written as

2.5 $$\begin{array}{rl} \frac{\partial \text{u'}}{\partial t}+\text{U}\cdot \nabla \text{u'}+\text{u'}\cdot \nabla \text{U}+\nabla p-\nu \nabla^{2}\text{u'} & \;=-{\left\langle {\text{u'}}_{l}\cdot \nabla {\text{u'}}_{l}+{\text{u'}}_{h}\cdot \nabla {\text{u'}}_{h}\right\rangle }_{l}\\ & \;\;-{\left\langle {\text{u'}}_{l}\cdot \nabla {\text{u'}}_{h}+{\text{u'}}_{h}\cdot \nabla {\text{u'}}_{l}\right\rangle }_{h} \end{array}$$

2.6 $$\begin{array}{r} \nabla \cdot \text{u'}=0, \end{array}$$

where we have written all linear terms on the left-hand side. This form is equivalent to those given in prior works (e.g. [4]), where separate equations are written for the low and high modes, respectively.

We non-dimensionalize lengths in terms of the gap width $d=R_{2}-R_{1}$ and velocities in terms of the inner cylinder velocity $U_{i}=R_{i}\Omega_{i}$. In our computations, the z-direction is periodic and thus $L_{z}$ is the periodicity length. We will also refer to the two Reynolds numbers ${Re}_{i}$, ${Re}_{o}$, related by

2.7 $$\frac{{Re}_{o}}{{Re}_{i}}=\frac{\mu }{\eta }.$$

It is also quite useful to characterize times in terms of the viscous diffusion time $\tau_{\nu }\equiv d^{2}/\nu$. In our units, $\tau_{\nu }={Re}_{i}$.

In our simulations, the possible spiral patterns are quantized by the periodicity in z and θ. Figure 3 shows that for ${Re}_{i}=700$, the pattern selected has $k_{z}=m=1$; ${Re}_{i}=900$ has the same pattern (not shown). Given this pattern, it is unclear *a priori* how to select the cut-off wavenumbers: unlike the case of plane Couette flow, where a self-sustaining process involving a quasi-linear transfer of streamwise waves to spanwise/wall-normal rolls is well established, here we have a turbulent structure that is bounded in both z and θ. Given this lack of apparent directionality, for all runs in this paper, $\Lambda =\Lambda_{z}=\Lambda_{\theta }$, though we note it would be interesting to consider models with varying $\Lambda_{\theta }$ but $\Lambda_{z}=N_{z}$, where $N_{z}$ is the DNS resolution. Such models are fully nonlinear in z and thus generalize the quasi-linear *restricted nonlinear* models used with success in shear flow turbulence [14–16].

We use the pseudo-spectral Dedalus framework (v. 2) [17] for all linear and nonlinear calculations. We discretize the three velocity components and pressure onto a basis of Chebyshev polynomials in the radial direction, while θ and z use Fourier bases. To evolve the solution forward in time, we use the implicit–explicit Runga–Kutta RK443 time stepper; linear terms are implicitly timestepped while nonlinear terms are explicit. Our initial value solver has been extensively tested for TC flow against the linear and nonlinear test cases in [18,19] including linear growth rates, nonlinear wave speeds and torque measurements; our spiral turbulence DNS results also agree with those in [12,20,21]. Resolutions and geometric parameters for all runs are given in table 1. We have confirmed our simulations are resolved by ensuring that the highest wavenumber components have spectral power at least $10^{5}$ smaller than the most energetic scale.

**Table 1.** Simulations.

${Re}_{i}$	${Re}_{o}$	η	Γ	$N_{r}$	$N_{\theta }$	$N_{z}$	$\Lambda_{\theta }$, $\Lambda_{z}$	notes
900	−3398	0.883	29.9	64	512	512	0, 1, 3, 5, 10	supercritical SPT
700	−3398	0.883	29.9	64	512	512	0, 1, 3, 5, 10, 20, 30, 60	subcritical SPT

Pseudospectra and critical parameters are calculated using eigentools [22].

## 3. Linear stability

**Figure 1.**
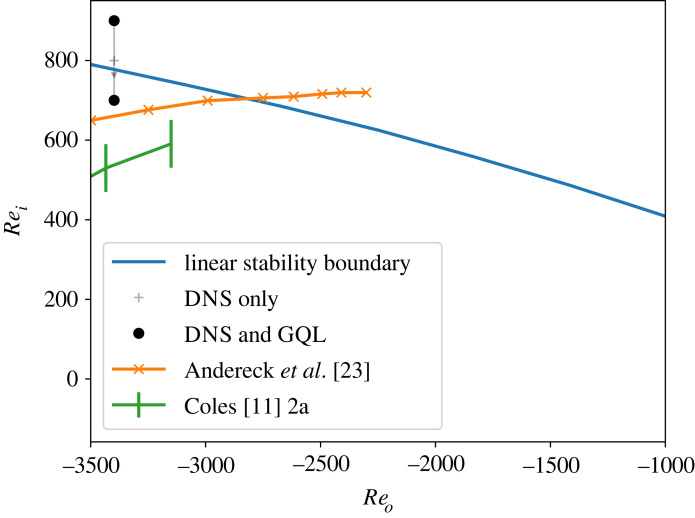
A summary of spiral turbulence runs in the ${Re}_{o}-{Re}_{i}$ plane showing linear stability boundary. Solid circles show points where we have conducted GQL analysis; crosses represent DNS steps along the path to subcritical behaviour. The curves labelled Andereck *et al.* [23] and Coles [11] give the boundaries of spiral turbulence hysteresis reported in those two papers. (Online version in colour.)

In order to determine the linear stability bound, we find the minimum ${Re}_{i}$ for a given ${Re}_{o}$ by solving the eigenvalue problem for equations (2.1). We note that for all ${Re}_{o}$ plotted in figure 1, the most unstable mode is never axisymmetric. At ${Re}_{o}=-3398$, where our nonlinear calculations are run, the most unstable mode has m=6 at onset. Figure 1 also shows the hysteresis boundaries given in references [11,23]. In both cases, original tabular data were not available, so we extracted the data from figs 2a of [11] and 16 of  [23], respectively, using WebPlotDigitizer [24]. The error bars on the Coles (1965) [11] data give the top and bottom of the markers from the original paper. We note in passing that the two studies do not agree on the lower boundary for hysteresis despite similar experimental geometries. Our subcritical experiment is within the more conservative hysteresis boundary given by Andereck *et al*. [23]. The spiral turbulence regime provides an ideal opportunity to test GQL in an environment with both spatio-temporal patterns and a tunable bifurcation: by choosing ${Re}_{i}$ and ${Re}_{o}$ appropriately, both subcritical and supercritical manifestations of spiral turbulence can be selected.

Following pioneering numerical work [12,25], we set the outer cylinder rotation rate to ${Re}_{o}=-3398$ and start a DNS run at ${Re}_{i}=900$, high enough to trigger spiral turbulence driven by linear instability from a low-amplitude, random initial condition satisfying ∇⋅u=0. From this seed run, we decrease ${Re}_{i}$ in steps shown in figure 1 where each point represents a DNS or GQL simulation run for one viscous time. The solid circles in figure 1 represent runs where we performed GQL analyses.

**Figure 2.**
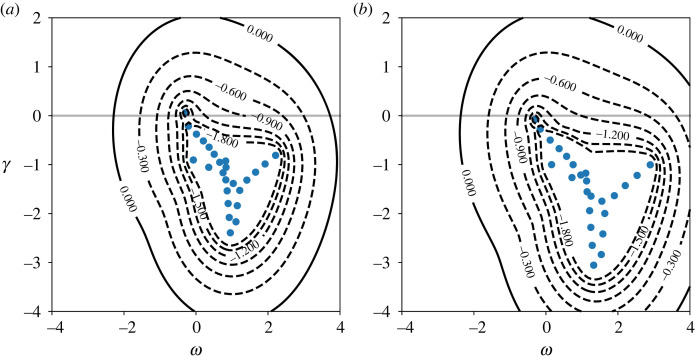
Spectra (blue points) and pseudospectra (contours of $\log_{10}\epsilon^{-1}$) for ${Re}_{i}=900$ (*a*) and ${Re}_{i}=700$ (*b*) for a mode with m=6 and $k_{z}=9.68$. The latter is the *z* wavenumber at the critical ${Re}_{i,\mathrm{crit}}≃778$. ω is the frequency and γ the growth rate at each point in the domain. The stability boundary is marked with a grey line at γ=0. The are seven equally spaced contours from −1.8 to 0. (Online version in colour.)

In order to demonstrate the effects non-normality plays, figure 2 shows both the modal spectrum (in blue points) and the ϵ−pseudospectrum for the supercritical and subcritical ${Re}_{i}$. The contour value $\log_{10}\epsilon^{-1}$ represents the set of values

3.1 $$\|{\left(z\text{I}-L\right)}^{-1}\|\geq \epsilon^{-1},$$

for a given complex number z=γ+iω. The spectrum σ are the eigenvalues corresponding to the temporal evolution of a linear solution, $e^{\sigma t}$ (see [26] for an introduction). Equation (3.1) depends on the choice of norm; here we use the standard energy norm. These contours in turn give a measure of the maximum amplification of a linear disturbance: the further negative contours extend into the upper-half plane, the larger a linear disturbance can grow as a result of non-normality, irrespective of the presence of instability. This linear effect is captured entirely by GQL (and QL, for that matter). However, the role it plays in the development of subcritical spiral turbulence remains an open question, as the relevant linear operator L in the subcritical case is not that associated with CCF U. Instead, any transient growth is generated by the saturated mean velocity profile.

## 4. Results

**Figure 3.**
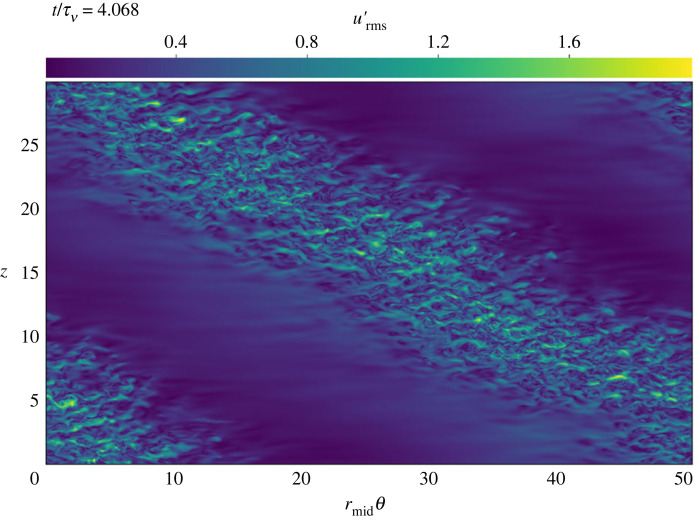
Slice of fluctuating RMS velocity in the middle of the radial domain of a DNS at ${Re}_{i}=700$, ${Re}_{o}=-3398$. This solution is well below the linear stability threshold at ${Re}_{i,\mathrm{crit}}≃778$. (Online version in colour.)

Figure 3 shows a typical DNS snapshot of the RMS velocity perturbation $u_{\mathrm{rms}}^{\prime }$ in the middle of the gap for ${Re}_{i}=700$. Despite being well below the linear stability threshold ${Re}_{i,\mathrm{crit}}≃778$, the flow morphology is nearly identical to the supercritical case ${Re}_{i}=900$ (not shown).

### (a) Supercritical spiral turbulence

**Figure 4.**
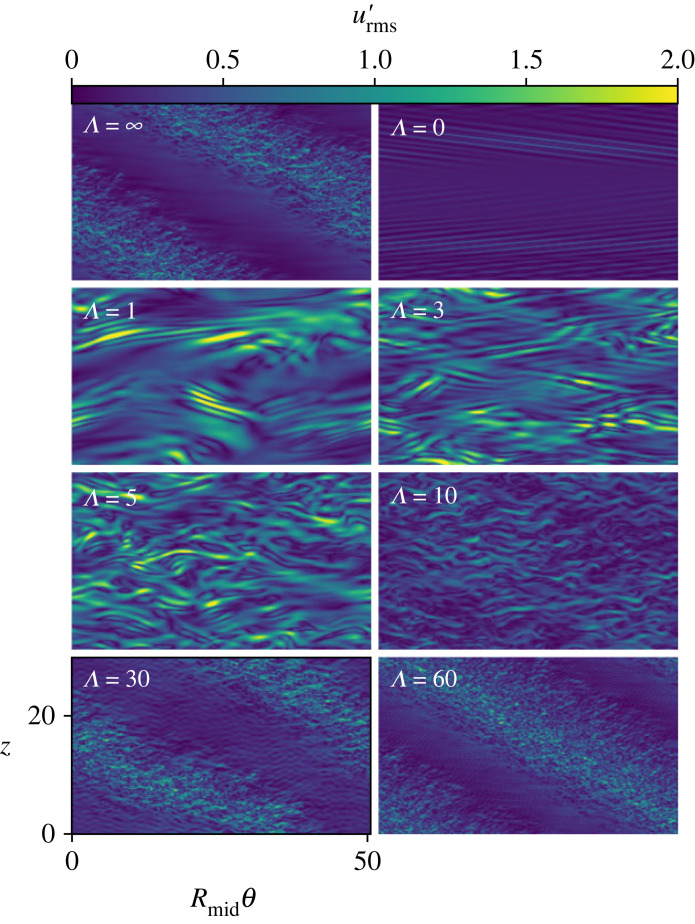
RMS velocity perturbations in the z−θ plane at $r=R_{\mathrm{mid}}$ for ${Re}_{i}=900,{Re}_{0}=-3398$. The upper left panel shows DNS, the upper right panel shows QL and the remaining six are GQL with Λ=1,3,5,10,30,60. Until Λ>30, spiral turbulence is not apparent. (Online version in colour.)

We first consider the supercritical case with ${Re}_{i}=900$. We initialize GQL simulations with Λ=0,1,3,5,10,30,60 using a snapshot of DNS data at $t≃1.5\tau_{\nu }$. Figure 4 shows slices at mid-gap, $r=R_{\mathrm{mid}}$. The first important thing to note is that low-order models are not able to maintain the spiral structure until Λ≥30. However, even including a single additional low mode, Λ=1, shows a significant increase in flow complexity compared with QL. It is quite interesting to note that QL *does* feature some degree of spatial intermittency—there are clearly separated regions hosting spiral waves interspersed with featureless, laminar patches.

**Figure 5.**
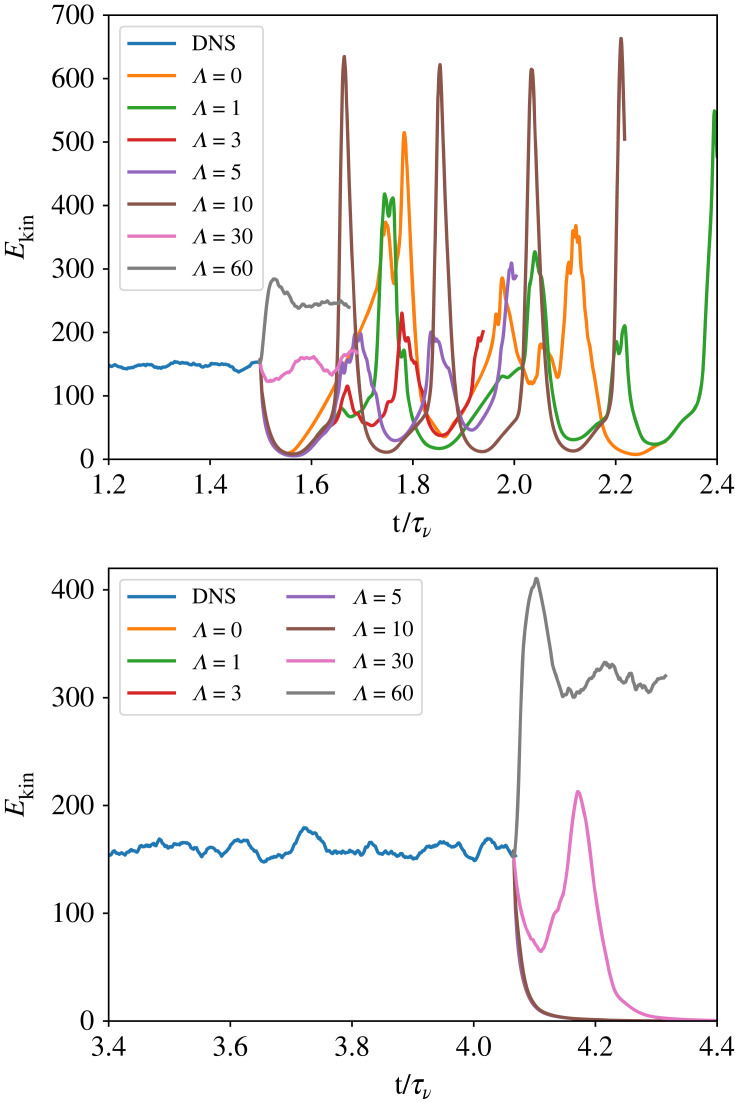
Perturbation kinetic energy as a function of time for ${Re}_{o}=-3398$. ${Re}_{i}=900$ (upper panel) is *above* the linear stability boundary. ${Re}_{i}=700$ (lower panel), continued by lowering ${Re}_{i}$ from previous simulations, is in the hysteretic region *below* the stability boundary. Starting from a saturated spiral turbulence state from DNS at $t/\tau_{\nu }≃1.5$ (upper panel), $t/\tau_{\nu }≃3$ (lower panel), we continue the solution with QL (Λ=0) and GQL with cut-offs Λ=1,3,5,10,30,60. At higher ${Re}_{i}$, all of the reduced models except Λ=60 begin high amplitude oscillations. At lower ${Re}_{i}$, Λ=60 is required to maintain the spiral turbulence state. (Online version in colour.)

Figure 5 shows the kinetic energy as a function of time and Λ for both the supercritical (upper) and subcritical (lower) runs. The supercritical case shows significant oscillations at all Λ<30. While both Λ=30 and 60 show spiral turbulence, Λ=30 has considerably more wave motion in the laminar region than either Λ=60 or DNS. Interestingly, figure 5 shows that Λ=30 has a *lower* kinetic energy much closer to that of DNS than does Λ=60.

**Figure 6.**
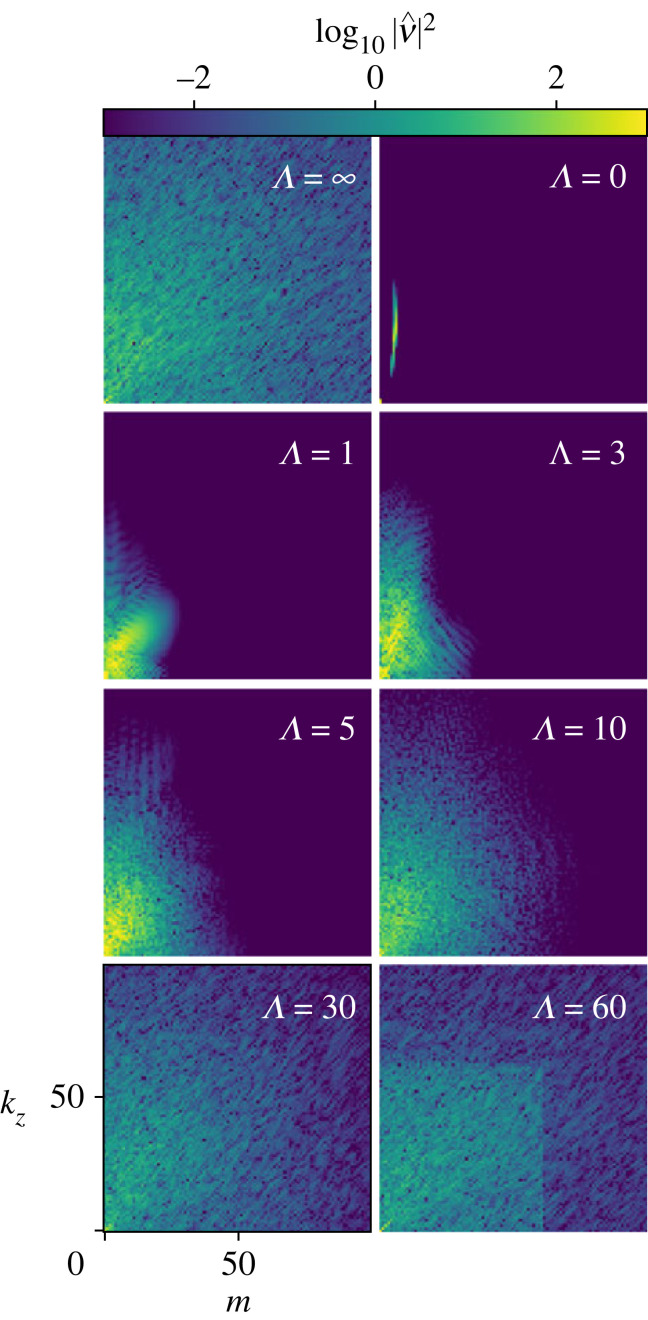
θ--z power spectra of v at mid-gap for ${Re}_{i}=900$ at various GQL cut-offs. Adding a single GQL mode significantly improves the power spectrum. Spiral turbulence appears at Λ=30. The obvious square in Λ=60 represents a sudden drop in spectral power at the cut-off wavenumber. The figure shows only the lowest 100 modes in each direction, though in the simulations $|k_{\max}|=256$ and $m_{\max}=511$. (Online version in colour.)

By examining power spectra in figure 6, we begin to see the reason for the more complex flows seen with Λ>0. Even low-order nonlinear scatter leads to a steady increase to higher k and m. The spectra of Λ=30 and 60 are of particular interest in understanding the nature of spiral turbulence: they show a transition toward filamentary structure aligned along the diagonal, very similar to DNS. The square feature clearly visible at Λ=60 is analogous to similar structures seen in channel flow turbulence (see fig. 4.20 of [6]). In that work, the author proposed a potential explanation in terms of the linear instability of the nonlinear solution: assume there is an instability of the mean $\left(m,k_{z})=(0,0\right)$ at some wavenumber $k^{\prime }$. If $k^{\prime }$ is counted among the high modes, high–low → high interactions smoothly populate the high modes. However, as Λ increases, it reaches a point at which the last unstable mode $k^{\prime }$ becomes counted among the low modes. Because low–low → high interactions are truncated in GQL, this results in a sudden decrease in the amount of energy available in the high part of the spectrum. Kellam [6] constructs a preliminary model of this behaviour for plane channel flow and finds support for the idea.

**Figure 7.**
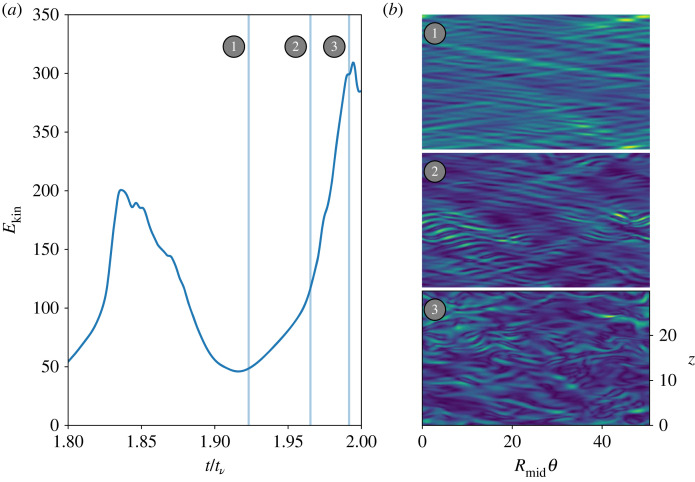
Kinetic energy (*a*) and flow morphologies (*b*) for ${Re}_{i}=900$, Λ=5 covering one full oscillation cycle. (*b*) Three times corresponding to the vertical lines in the kinetic energy plot on (*a*). At the kinetic energy minimum (point 1), the flow shows a IPS-like morphology, while an INT burst occurs at the knee (point 2), and a more space-filling turbulent state occurs at maximum energy (point 3). (Online version in colour.)

Focusing on Λ=5, figure 7 shows that the flow goes through a series of different states bearing a strong resemblance to other well-known TC flow patterns originally identified in an exhaustive series of experiments reported in [18] and subsequently found in simulations (e.g. [12]). At the time of kinetic energy minimum, labelled 1 in the left-hand side of figure 7, the flow seems to recapitulate the interpenetrating spirals characteristic of lower Re counter-rotating flows. As the kinetic energy rises, reaching a knee at point 2, the flow morphology resembles the patchy bursts characteristic of the intermittency regime. Finally, at the peak of the kinetic energy oscillation, point 3 in figure 7, the flow reaches its closest approach to something resembling spiral turbulence, though it is very difficult to identify the flow as such from a slice at a single point in time and plane in space.

**Figure 8.**
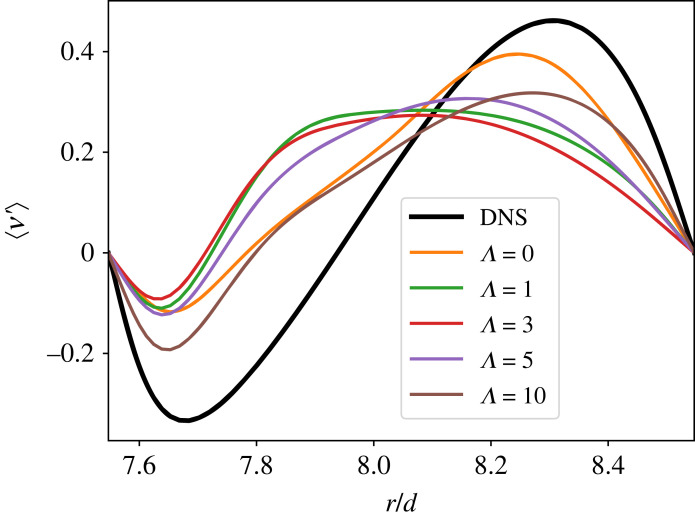
Mean deviation from CCF for ${Re}_{i}=900$, ${Re}_{o}=-3398$. The heavy line is DNS, the orange line is QL, and all others are GQL with Λ=1,3,5,10. (Online version in colour.)

This is notable for a few reasons. At ${Re}_{i}=900,{Re}_{o}=-3398,$ the system is not in a hysteretic region of parameter space. Thus, we do not expect this behaviour to be due to GQL picking up other, coexisting solutions. It also highlights the delicate balance an intermittent laminar/turbulent solution represents. The turbulence in this solution has a very broad range of scales, as evidenced by figure 3. Understanding how it exchanges energy with the mean flow is crucial to understanding both the saturation of the underlying linear instability as well as the maintenance of the nonlinear state. Finally, in figure 8, we show the azimuthal velocity averaged over θ and z, giving the mean deviation from CCF as a function of GQL cut-off Λ. Note that no conditional averaging [20] is performed here, so this represents an average over both laminar and turbulent regions for DNS. As Λ is increased, the profile approaches the DNS case; this is typical of GQL behaviour (fig. 7 of [4] shows the same for rotating plane Couette flow). However, QL performs slightly better than low-order GQL with Λ<5.

### (b) Subcritical spiral turbulence

In order to study subcritical spiral turbulence, we ran a series of DNS with decreasing ${Re}_{i}$. Each is initialized with the last timestep of the prior run and run for one viscous time. Once we reached ${Re}_{i}=700$, we run a DNS for a final viscous time and then repeat the procedure from the supercritical GQL study. The lower panel of figure 5 shows that all Λ<30 revert to the laminar state within $0.1\tau_{\nu }$. At Λ=30, the solution recovers from the decay and begins to climb in energy again but ultimately returns to the laminar CCF state. Curiously, our Λ=60 run not only remains in a non-laminar solution for as long as we have been able to run it for, it also correctly reproduces the spiral turbulence pattern, just as in the supercritical case.

The ability of GQL to reproduce spiral turbulence in the subcritical regime is quite significant. First, while Λ=60 corresponds to the fully nonlinear interactions occurring in a rather large fraction of the total number of modes in the simulation, it is important to note that a DNS at $n_{\theta }=n_{z}=60$ would be massively underresolved, possibly numerically unstable. Second, the ability of nonlinear interactions to sustain turbulence where non-normal linear dynamics alone cannot suggests an important role for exact coherent states in subcritical TC flow. Because GQL retains all linear dynamics and low-order nonlinear couplings, in addition to the couplings between the mean flows and high modes, if non-normal dynamics played a strong role in subcritical turbulence, one would expect the behaviour seen at Λ=30 to lead to sustained, rather than transient turbulence.

## 5. Conclusion

We have presented a series of simulations demonstrating the performance of GQL at various cut-offs for TC flow in the spiral turbulence regime. GQL is a significant improvement over QL models in representing statistical quantities, particularly the power spectra. However, it does not retain an unambiguous spiral form in the supercritical regime until Λ≃30; at subcritical ${Re}_{i}$, Λ≃60 is required to see any sustained flows at all. Interestingly, when GQL solutions do sustain non-CCF flows in the subcritical regime, they also produce spiral patterns. Prior work has focused on the utility of GQL in presenting a more accurate *statistical* picture of turbulent flows than QL [3–6]. This focus is particularly important in light of its potential as a means of improving the accuracy of direct statistical simulation, a method for studying turbulence by computing its statistics directly, rather than individual realizations [2]. We have shown here that it is possible to use GQL as a means to investigate the nonlinear interactions of greatest importance for maintaining the intricate patterns manifested in TC flow. In future work, we will consider the ability of GQL to maintain exact coherent states identified by Deguchi *et al.* [27], and the dynamics of which were studied recently by Wang and co-workers [28,29]. Understanding how these interactions work to shape subcritical TC turbulence represents a promising new avenue to understanding how ECS are maintained and how they in turn sustain turbulence below linear stability bounds. In particular, one can begin to understand the relative importance of non-normal effects (represented entirely with GQL) and nonlinear ECS.

## Data Availability

All code for nonlinear initial value problems, linear eigenvalue and non-modal stability analyses, and plotting are available at https://github.com/jsoishi/GQL_TC. Dedalus version 2 is available at https://github.com/DedalusProject/dedalus/tree/v2_master. eigentools is available at https://github.com/DedalusProject/eigentools. Simulation data are available upon request.
